# Impact of Ag on the Limit of Detection towards NH_3_-Sensing in Spray-Coated WO_3_ Thin-Films

**DOI:** 10.3390/s22052033

**Published:** 2022-03-05

**Authors:** Anusha, Aninamol Ani, P. Poornesh, Albin Antony, Bhaghyesh, Igor V. Shchetinin, K. K. Nagaraja, Saikat Chattopadhyay, K. B. Vinayakumar

**Affiliations:** 1 Department of Physics, Manipal Institute of Technology, Manipal Academy of Higher Education, Manipal 576104, Karnataka, India; anusha.dpas2@learner.manipal.edu (A.); aninamol.ani@learner.manipal.edu (A.A.); bhaghyesh.mit@manipal.edu (B.); nagaraja.kk@manipal.edu (K.K.N.); 2 Department of Surface and Plasma Science, Faculty of Mathematics and Physics, Charles University, V Holešovičkách, Praha 8, 218000 Praha, Czech Republic; albin.antony@matfyz.cuni.cz; 3 Department of Physical Materials Science, National University of Science and Technology “MISiS”, Leninskiy Pr. 4, 119049 Moscow, Russia; ingvar@misis.ru; 4 Department of Physics, School of Basic Sciences, Manipal University Jaipur, Jaipur 303007, Rajasthan, India; saikat.chattopadhyay@jaipur.manipal.edu; 5 Micro and Nano Fabrication Department, INL-International Iberian Nanotechnology Laboratory Avenida Mestre Jose Veiga, 4715-330 Braga, Portugal; vinaya.basavarajappa@inl.int

**Keywords:** NH_3_ sensing, oxygen vacancies, WO_3_ films

## Abstract

Ag-doped WO_3_ (Ag–WO_3_) films were deposited on a soda-lime glass substrate via a facile spray pyrolysis technique. The surface roughness of the films varied between 0.6 nm and 4.3 nm, as verified by the Atomic Force Microscopy (AFM) studies. Ammonia (NH_3_)-sensing measurements of the films were performed for various concentrations at an optimum sensor working temperature of 200 °C. Enrichment of oxygen vacancies confirmed by X-ray Photoelectron Spectroscopy (XPS) in 1% Ag–WO_3_ enhanced the sensor response from 1.06 to 3.29, approximately 3 times higher than that of undoped WO_3_. Limit of detection (LOD) up to 500 ppb is achieved for 1% Ag–WO_3_, substantiating the role of Ag in improving sensor performance.

## 1. Introduction

Various types of gas sensors, including chemoreceptive-, ionization-, acoustic-, and resonant-based sensors are gaining more attention in different sectors, such as environmental monitoring, food safety, medical diagnosis, and industrial applications [1,2,3]. Among these, chemoreceptive-based metal oxide semiconductors (MO_x_) have received tremendous interest in gas sensing due to their high surface/volume ratio, as the gas reaction process is a surface phenomenon. Different MO_x_, such as ZnO, SnO_2_, WO_3_, and TiO_2_ [4,5] have been extensively studied for gas-sensing applications. Tungsten oxide (WO_3_) has emerged as a potential MO_x_ to detect the gases, such as NH_3_, H_2_, NO_2_, CO, and alcohol vapors, because of their inherent properties, including excellent electrical conductivity, sensitivity, and selectivity [6,7]. To enhance the sensing performance further, doping is considered one of the possible approaches. Transition metal doping, such as Cr, Cu, Ag, and Pd, would induce the defects causing enrichment of oxygen vacancies via hole compensation mechanism. These metals act as promoters and increase the sensing properties via spill-over effect or Fermi-level mechanisms [8,9,10]. Godbole et al. [11] reported the NH_3_-sensing properties of Pd/WO_3_ films with the response of 0.27 at a working temperature of 225 °C. Lu et al. [12] presented studies on NO_2_-sensing properties of Ag–WO_3_ nanoparticles and reported high sensitivity and better selectivity. Xu et al. [13] obtained a superior sensor response for Ag–WO_3_ core-shell nanostructures towards alcohol vapor at a working temperature of 340 °C. All these studies have shown that metal incorporated into WO_3_ has enhanced the sensor performance.

In this regard, we have illustrated the NH_3_-sensing performance of Ag–WO_3_ films via the spray pyrolysis deposition technique. Ag is a noble metal that enhances the sensor response of WO_3_ due to electronic sensitization mechanism [14]. Due to large area deposition, ease of operation, and cost effectiveness, spray pyrolysis is chosen to deposit films in this report. The availability of literature is scarce on Ag–WO_3_ for NH_3_ sensing, and one of the works published is on hydrothermal technique [14]. To the best of our knowledge, no reports on spray-deposited Ag–WO_3_ films are accessible for NH_3_ sensing. Therefore, our attempt has proved the possibility of these films for NH_3_ sensing by spray pyrolysis. Though the literature is available on NH_3_ sensing by metal-doped WO_3_, the majority of the sensors work at high temperatures (>250 °C) and the reported detection limit is high [14,15,16]. Ammonia (NH_3_), a reducing gas, is a major pollutant from the automobiles, fertilizer, and mining industries. According to the OSHA (Occupational Health and Safety Administration) report, the permissible limit for NH_3_ is 35 ppm for 15 min [17]. High exposure to the gas for a long time will trigger lung- and kidney-related diseases, causing incurable damage to human health. In this context, we aimed to lower the operating temperature and detection limit, which are essential for the sensing applications. Thus, in the current work, we have reported spray-pyrolyzed Ag–WO_3_ films as a promising candidate for NH3 sensing at a working temperature of 200 °C with the detection limit of 500 ppb.

## 2. Experimental

Undoped and silver-doped tungsten oxide films (Ag–WO_3_) were synthesized by the spray pyrolysis method. For undoped WO_3_, ammonium metatungstate hydrate (99.99% purity) is dissolved in double-distilled water and a homogeneous solution is obtained. For Ag doping, silver nitrate was taken as a precursor, and doping was done at a 1 wt.%, 3 wt.%, and 5 wt.% ratio. Solution concentration was maintained at 0.01 M. Deposition parameters, such as substrate temperature and flow rate, were kept constant at 400 °C and 1 mL/min, respectively.

**Figure 1:**
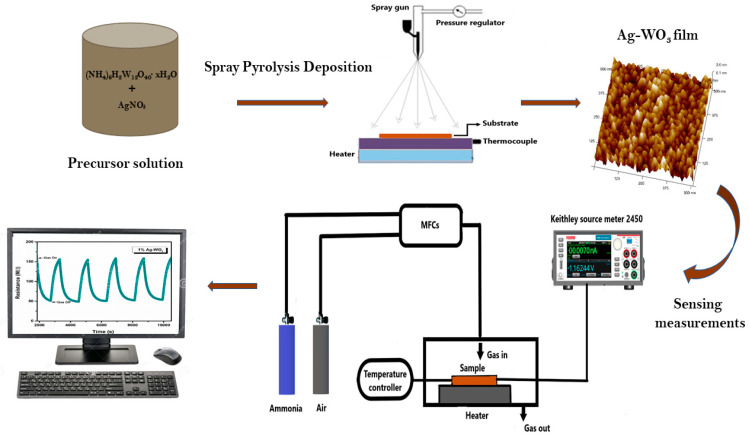
Schematic illustration of film deposition and ammonia sensing measurements of Ag–WO_3_.

Crystal structure and phase identification were performed via Rigaku SmartLab X-ray diffractometer with Cu Kα radiation at 40 kV, 30 mA. Raman analysis was performed using a Horiba JOBINYVON LabRAM HR spectrometer for the confirmation of structure. Morphological studies were performed via Innova SPM Atomic Force Microscope (AFM). AXIS ULTRA X-ray Photoelectron Spectroscope is used for oxidation state and composition studies. Gas sensing was conducted via dc probe measurements in an enclosed chamber (2.96 × 10^4^ cm^3^ by vol.) by purging synthetic air (79% N_2_ + 21% O_2_) and NH_3_ to the sample. The flow of gas was controlled using programmable mass flow controllers (MFCs), and the overall flow was kept constant at 500 sccm. I-V measurements were performed via Keithley source meter 2450 using silver paste electrodes. Sensor response of the films was evaluated using the equation, ($\frac{R_{a}-R_{g}}{R_{g}}$). R_a_ and R_g_ suggest the film resistance in air and target gas (NH_3_), respectively. Schematic representation of film synthesis and ammonia-sensing measurements of Ag–WO_3_ film is represented in the Figure 1.

## 3. Results and Discussion

### 3.1. Structural and Morphological Analysis

**Figure 2:**
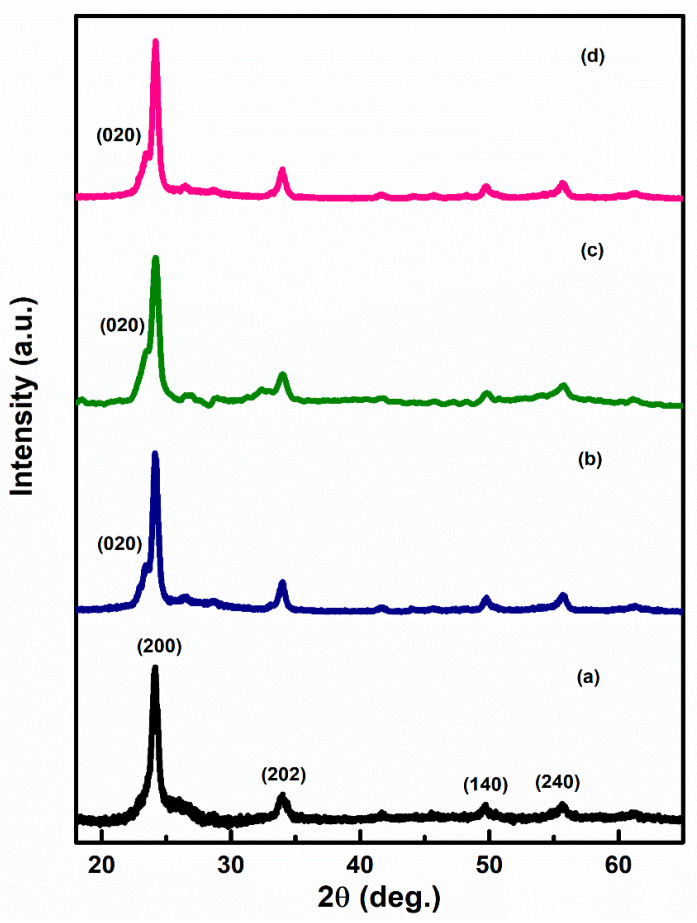
XRD pattern of (**a**) 0%, (**b**) 1%, (**c**) 3%, and (**d**) 5%, Ag–WO_3_ films.

Figure 2a–d represents the XRD pattern of Ag–WO_3_ films at varied Ag doping levels. Diffraction peaks at the angles 24.1°, 33.9°, 49.6°, and 55.6° correspond to (200), (202), (140), and (240) planes, indicating monoclinic phase (γ-WO_3_) of WO_3_ films (JCPDS card no. 43–1035) [12,18]. The appearance of sharp diffraction peaks implies that the deposited films have high crystallinity. The absence of any further peaks in the spectra endorsed the non-existence of any impurity phase in the prepared Ag–WO_3_ films. All the films exhibited ‘a’ axis preferential orientation, i.e., along (200) plane. Evolution of (020) peak centered at 23.4° is observed upon addition of Ag into WO_3_. The introduction of Ag formed new nucleating centers for WO_3_, ultimately retaining monoclinic structure. The crystallite size of the films was determined by employing the Scherrer formula [19] and it varies from 11.5 nm to 14.7 nm. The dislocation density and microstrain [19] were also evaluated and are presented in Table 1.

**Figure 3:**
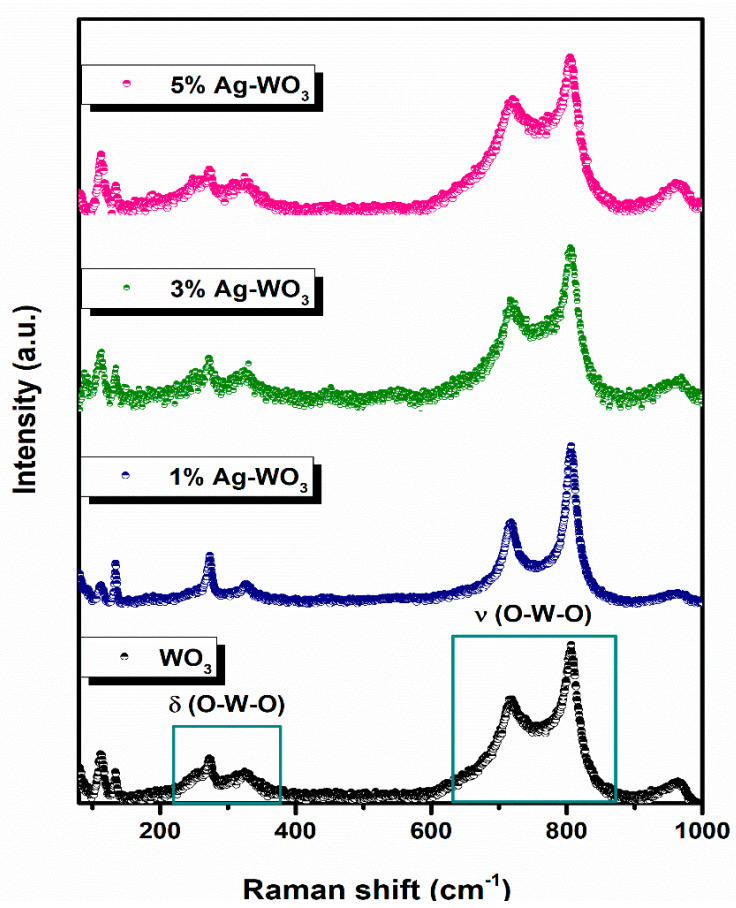
Raman spectra of Ag–WO_3_ films.

Since triclinic and monoclinic phases of WO_3_ exhibit the same set of XRD peaks, we have conducted Raman measurements for further confirmation [20,21]. Figure 3 illustrates the Raman spectra of Ag–WO_3_ films excited with the 532 nm laser source. Clear visibility of peaks at ~112 cm^−1^, ~134 cm^−1^, ~270 cm^−1^, ~325 cm^−1^, ~719 cm^−1^, ~806 cm^−1^, and ~963 cm^−1^, attributed to the different modes of vibrations in the WO_3_ lattice. Peaks corresponding to ~719 cm^−1^ and ~806 cm^−1^ represent the asymmetric and symmetric stretching vibrational modes (ν_as_ and ν_s_) of O–W–O bonds and these are often referred to as the strongest monoclinic WO_3_ modes [22]. Peaks located at ~270 cm^−1^ and ~325 cm^−1^ attribute to the O–W–O bending vibrational modes (δ) and peaks below 200 cm^−1^ contribute to the lattice vibrational modes [23]. All the films exhibit a peak at ~963 cm^−1^, which can be ascribed to the symmetric stretching vibration of terminal W=O bonds possibly associated with the clusters on the film surface [24,25]. The frequency of vibration of the W=O bond is predicted to be higher than that of the W–O bond since the W–O single bond is weaker than the W=O double bond. Raman measurements confirmed the monoclinic phase of Ag–WO_3_ films.

**Figure 4:**
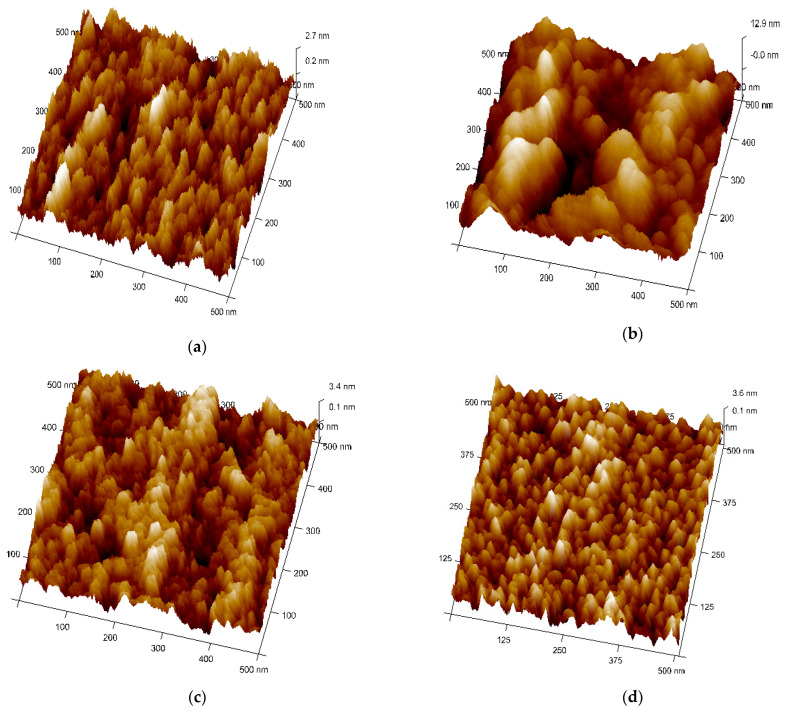
Topographical view of Ag–WO_3_ films at (**a**) 0%, (**b**) 1%, (**c**) 3%, and (**d**) 5% Ag concentrations.

Figure 4a–d shows the topography and microstructures (3D view) of Ag–WO_3_ films examined by AFM in tapping mode configuration. All the samples were scanned in the area of 0.5 × 0.5 µm^2^. Figure 4b represents the topographical view of 1% Ag–WO_3_. It suggests the coalescence of grains upon Ag incorporation forming larger globules of particles with the presence of voids. At 3% and 5%, Ag doping (Figure 4c,d), well-separated smaller grains are visible with almost uniform distribution. RMS surface roughness of the films was evaluated using NanoScope Analysis software and given in Table 1. Upon Ag doping, the roughness value of the films varied, and for 1% Ag–WO_3_ higher value of roughness was recorded.

### 3.2. XPS Studies

**Figure 5:**
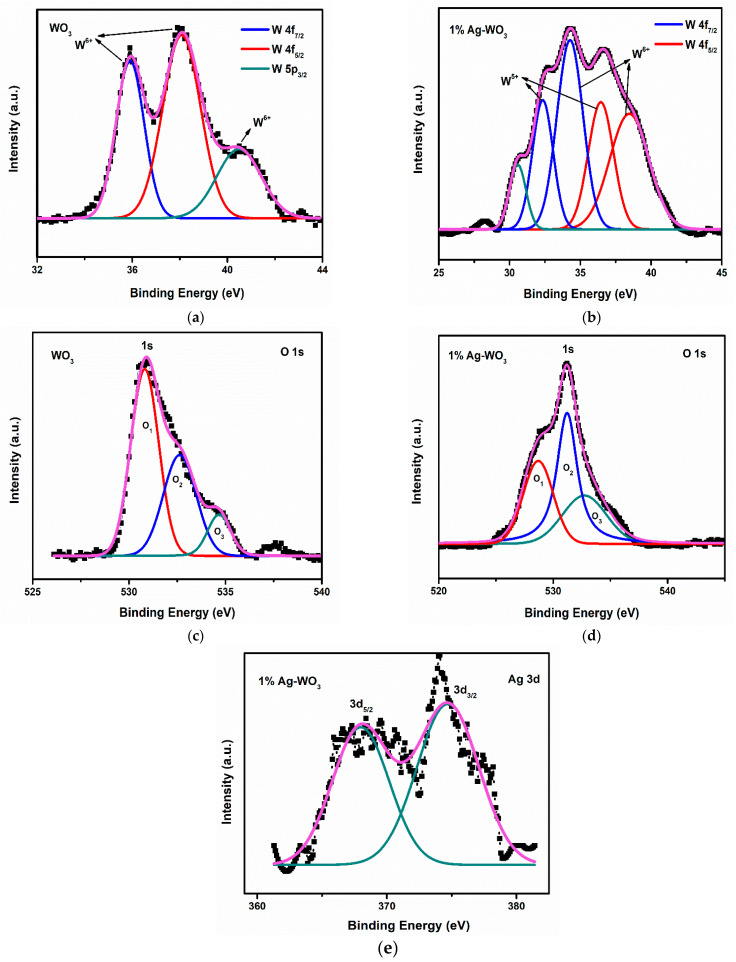
(**a**–**e**): XPS spectra of W, O, and Ag core levels in WO_3_ and 1% Ag–WO_3_ films.

Elemental composition and chemical state of Ag–WO_3_ films (at 0% and 1% Ag conc.) were examined via the XPS technique. Figure 5a shows the deconvoluted spectra of W 4f split into spin-orbit doublet, namely W 4f_7/2_ and W 4f_5/2_ located at the binding energies (E_b_) of 35.9 eV and 38.1 eV, respectively, in undoped WO_3_ film. These represent the +6 oxidation state of W and another satellite peak at 40.5 eV indicates the W 5p_3/2_ component corresponding to the same oxidation state [26]. The incorporation of Ag into WO_3_ has led to the formation of +6 and +5 oxidation states of W as depicted in Figure 5b. Deconvolution of W 4f in Figure 5b has resulted in two pairs of doublets viz. one at 34.2 eV and 38.4 eV equivalent to W 4f_7/2_ and W 4f_5/2,_ respectively, for the +6 oxidation state of tungsten, and other pairs of doublets comprised of W 4f_7/2_ and W 4f_5/2_ centered at 32.4 eV and 36.5 eV are comparable to the +5 oxidation state of tungsten [27]. The peak positioned at 30.6 eV was assigned to the metallic tungsten [28]. If oxygen vacancy is present, electron density near neighboring W atoms intensifies, causing higher screening of its nucleus and consequently, the 4f energy level is predicted to be at lower E_b_ [23,27]. In the present studies, the shoulder associated with the W 4f is generated as a result of electrons emitted from W atoms near oxygen vacancies, and hence W atom has an oxidation state less than +6, resulting in the formation of sub-stoichiometric WO_3−x_.

The deconvolution of O 1s spectra has produced 3 peaks (O_1_, O_2_, and O_3_) in both WO_3_ and 1% Ag–WO_3_ films (Figure 5c,d) respectively. O_1_ centered at E_b_ of 530.8 eV and 528.7 eV in WO_3_ and 1% Ag–WO_3_ respectively, was ascribed to O^2−^ in the lattice. Similarly, O_2_ centered at 532.6 eV and 531.2 eV denote the lattice oxygen associated with the oxygen-deficient regions near W ions, which are generally referred to as oxygen vacancies (V_o_) [29,30]. Area ratio of oxygen vacancies is estimated from peak area calculations as given below:

$${\%V}_{o}=\frac{O_{2}}{\left(O_{1}+O_{2}+O_{3}\right)}\times 100$$

V_o_ for WO_3_ and 1% Ag–WO_3_ was found 35% and 45%, respectively. Hence, the studies inferred that oxygen vacancies increased by 10% in 1% Ag–WO_3_. The least intense peaks centered at 534.7 eV and 532.8 eV, respectively in WO_3_ and 1% Ag–WO_3_, connected to the adsorbed oxygen species ($O_{2}^{-}$, ${\mathrm{OH}}^{-}$) [30]. Figure 5e represents the characteristic peaks of Ag at E_b_ of 368 eV and 374.5 eV contributes to Ag 3d_5/2_ and Ag 3d_3/2_, respectively, in 1% Ag–WO_3_. These peaks are assigned to the Ag^0^/Ag^1+^ states of silver [28,31].

### 3.3. Gas Sensing Properties

**Figure 6:**
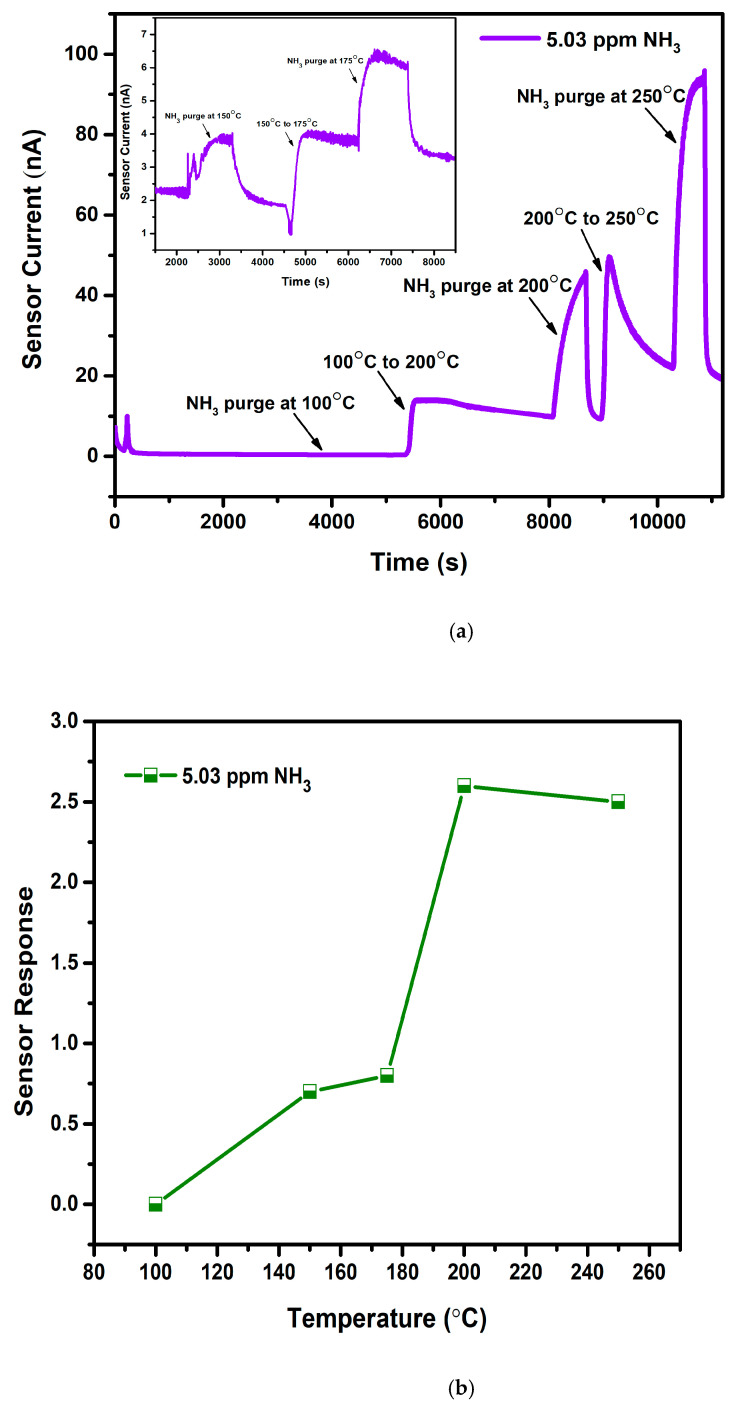
(**a**): Temperature optimization plot of WO_3_ film at 100 °C, 200 °C, and 250 °C (Inset: At 150 °C and 175 °C). (**b**): Sensor response vs. temperature plot for WO_3_ film.

Operation temperature for WO_3_ towards NH_3_ was fixed by purging gas at various temperatures and calculating subsequent sensor response as presented in Figure 6a,b. Figure 6a represents the obtained graph for sensor current versus time wherein 5.03 ppm NH_3_ is purged at 5 different temperatures: 100 °C, 150 °C, 175 °C 200 °C, and 250 °C. An increase in temperature causes an increase in the sensor current, demonstrating the semiconducting nature of the WO_3_ films. At 100 °C, the graph shows a straight line, denoting no response for NH_3_ due to the low thermal energy of the gas required for the chemisorption process. Low response was noted at 150 °C and 175 °C. An increase in the temperature to 200 °C has shown maximum response, and thereafter the response has decreased as indicated in Figure 6b. With the enhancement in the temperature, WO_3_ started to respond, as enough thermal energy is provided for the surface reaction to occur by overcoming the activation energy barrier [32]. After the maximum response, a reduction in the sensing performance at 250 °C is due to the lower adsorption capability of gas molecules. In addition, it is observable that baseline stability was lost above 200 °C (Figure 6a). Hence, 200 °C is considered a suitable operating temperature for all the deposited films, and further studies were conducted at the same temperature.

**Figure 7:**
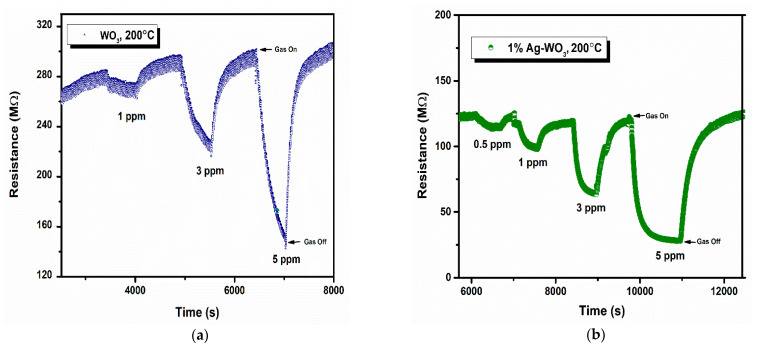

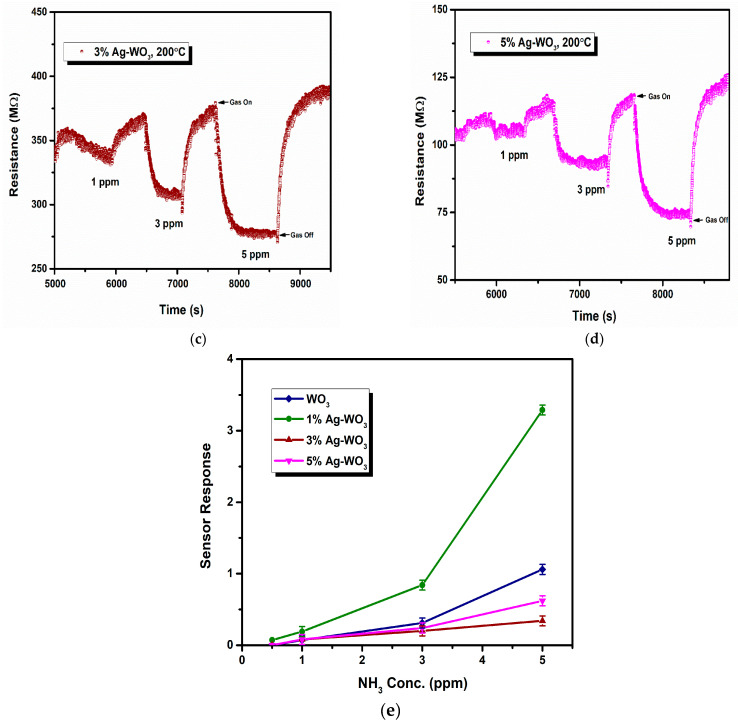
(**a**–**d**): Transient response curves of Ag–WO_3_ films and (**e**) sensor response of the films at various NH_3_ concentrations with error bars.

Sensor response, rate of response, and recovery ($\tau_{res}\;\mathrm{and}\;\tau_{rec\;}$) are the essential parameters that determine the efficiency of any sensor. Figure 7a–d depicts the transient response curves of Ag–WO_3_ films (0%, 1%, 3% and 5% Ag conc.) for different NH_3_ concentrations. Figure 7e shows the variation of sensor response with the NH_3_ concentration. The sensor response for 5 ppm NH_3_ was found to be 1.06, 3.29, 0.34, and 0.62 with the standard error of ±0.07 for undoped, 1%, 3%, and 5% Ag-doped WO_3_ films, respectively. The highest response was recorded for 1% Ag–WO_3_ with the $\tau_{res\;}$ of 5.5 min and $\tau_{rec}$ of 7.9 min. Doping of Ag reduced the detection limit from 1 ppm to 0.5 ppm (500 ppb) in 1% Ag–WO_3_. ‘Ag’, being a noble metal, acts as a catalyst due to chemical sensitization and also causes electronic sensitization due to the interaction between Ag and WO_3_, which might have induced the enhancement in the sensor response of the WO_3_ film [9]. AFM studies revealed that 1% Ag–WO_3_ showed higher surface roughness value (Table 1) compared to other Ag concentrations. Surface roughness is one of the parameters that can increase the surface to volume ratio of the films and thereby enhances the gas adsorption capacity [33]. M. Kumar et al. [34] and J. M. Lee et al. [33] observed similar results for CO and H_2_-gas sensing, respectively. Augmentation in the sensing performance of 1% Ag–WO_3_ could be accredited to improved oxygen vacancies upon Ag doping, which is confirmed by XPS analysis and also higher surface roughness and void formation on the film surface is proven by AFM, providing a greater number of adsorption centers for NH_3_ [22,35]. Nevertheless, a decrease in sensor response beyond 1% Ag doping is connected to lower surface roughness of the films and catalytic efficiency of Ag, indicating that while Ag inclusion improves sensing performance, increasing the doping concentration above the optimum level may reduce catalytic efficiency [27]. Also, XPS revealed the possibility of Ag_2_O formation upon Ag doping. When Ag concentration exceeds 1%, Ag_2_O amounts might increase and cause an increase in the depletion layer width, deteriorating the sensor response of the films.

**Figure 8:**
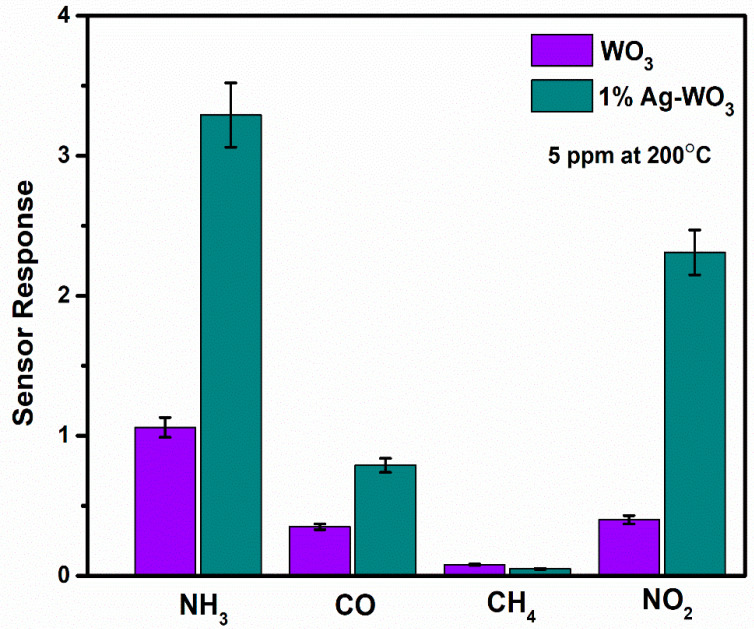
Representation of selectivity studies for WO_3_ and 1% Ag–WO_3_ with error bars.

**Figure 9:**
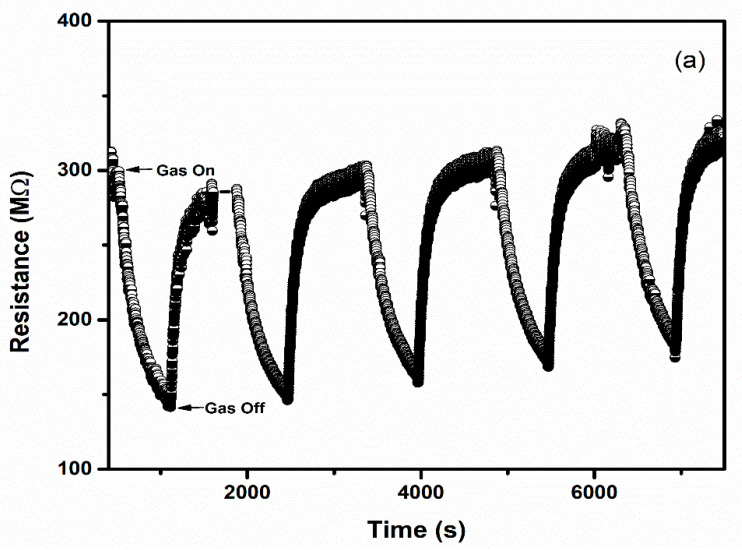

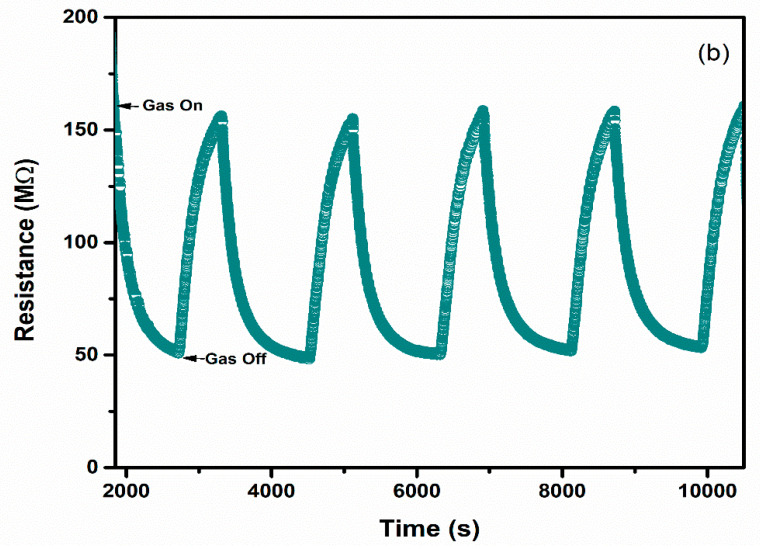
Repeatable sensing characteristics of (**a**) WO_3_ (**b**) 1% Ag–WO_3_ at 5 ppm NH_3_ concentration.

Selectivity and repeatability are the key factors that decide the performance of the sensors. Figure 8 represents the bar graph elucidating sensor responses of WO_3_ and 1% Ag–WO_3_ films towards various gases. These films are tested at a 5-ppm concentration towards NH_3_, CO, CH_4,_ and NO_2_. Both films showed the highest response to NH_3_ indicating the selective nature of the deposited films towards ammonia among other gases. Repeatability measurements of about 5 cycles were performed for both WO_3_ and 1% Ag–WO_3_ films, shown in Figure 9a,b. Almost repeatable sensing characteristics were obtained 5 times for NH_3_ purge at 5 ppm concentration, implying the stable response of the films. In comparison, sensing properties of the current work with the previously reported literature is given in Table 2. The WO_3_ nanoflakes synthesized via spray pyrolysis detected the lowest NH_3_ concentration to be up to 120 ppm at 150 °C [36], while the V-WO_3_ films synthesized by soft chemical route exhibited a detection limit of 100 ppm towards NH_3_ at 300 °C [37]. The Cr–WO_3_ nanosheets synthesized by acidification with impregnation process detected 2 ppm NH_3_ at 400 °C [38], whereas the WO_3_–Fe_2_O_3_ nanocomposites by hydrothermal synthesis demonstrated NH_3_ detection of 25 ppm at 300 °C [39]. In the present work, we were able to achieve lowest detection limit of 500 ppb towards NH_3_ gas, keeping an operating temperature of 200 °C, which is a significant improvement in the sensing performance compared to the literature presented in Table 2.

**Figure 10:**
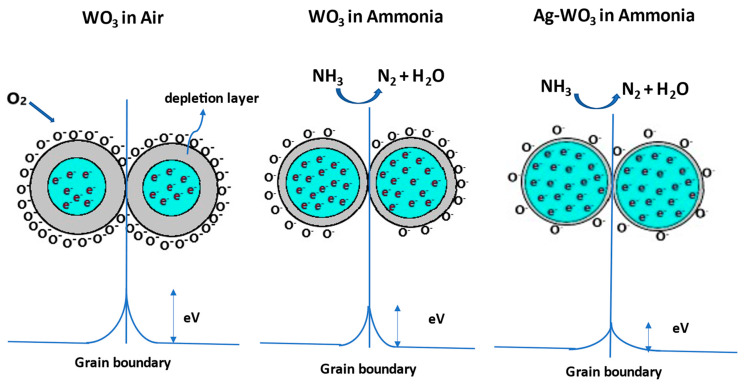
Sensing mechanism involved between the grains of WO_3_ and Ag–WO_3_ films.

The well-known gas detection mechanism of metal oxide gas sensors involves the resistance variations caused by the chemisorption of gas molecules on the sensor surface. Depending on the operating temperature, WO_3_ exposure to the synthetic air produces molecular/atomic oxygen ions ($O_{2}^{-},O^{-},{\mathrm{and}\;O}^{2-}$). Electron transfer from the surface of the WO_3_ to the adsorbed oxygen species creates a band bending region called the depletion layer, resulting in a decrease in the carrier concentration of the film and an increase in the resistance. Later, when WO_3_ is subjected to NH_3_ exposure, the depletion layer width decreases due to the release of electrons back to the WO_3_. As Ag–WO_3_ is exposed to NH_3_, the depletion layer further decreases due to the electronic sensitization mechanism giving rise to an increment in carrier concentration and a reduction in surface resistance. Figure 10 illustrates the schematic representation of the sensing mechanism. Specific reactions involved in the process are governed by the equations given below [14,35]:

(1) $$O_{2\left(\mathrm{gas}\right)}\to O_{2\left(\mathrm{ads}\right)}$$

(2) $$O_{2\left(\mathrm{ads}\right)}{}^{}+e^{-}\to O_{2}^{-}$$

(3) $$O_{2}^{-}+e^{-}\to 2O^{-}$$

(4) $$4{\mathrm{NH}}_{3}(g)+5O_{2}^{-}\to 4\mathrm{NO}+6H_{2}O+5e^{-}$$

(5) $$2{\mathrm{NH}}_{3}(g)+3O^{-}\to N_{2}+3H_{2}O+3e^{-}$$

## 4. Conclusions

Spray pyrolyzed Ag–WO_3_ films were investigated for NH_3_ sensing at different concentrations. XRD spectra depicted the monoclinic phase of deposited films, which was further verified by Raman analysis. Oxygen vacancies, higher surface roughness, and void-like structures of 1% Ag–WO_3_ contributed to enhancement in the sensor response value. Selectivity studies of WO_3_ and 1% Ag–WO_3_ towards NH_3_, NO_2_, CH_4,_ and CO exhibited an excellent response to NH_3_ compared to other gases. Spray deposited Ag–WO_3_, as a unique approach for NH_3_ sensing, produced a superior response at a low operating temperature of 200 °C with a detection limit in the sub-ppm levels.

## Figures and Tables

**Table 1:** Structural and morphological parameters of Ag–WO_3_ films.

Ag Conc. (wt.%)	2θ, (200)	Crystallite Size D (nm)	Dislocation Density δ (×10^15^ m^−2^)	Microstrain ε (×10^−3^)	RMS Surface Roughness (nm)
0	24.12°	13.0	5.9	2.7	0.8
1	24.16°	13.6	5.4	2.5	4.3
3	24.14°	11.5	7.5	3	1.1
5	24.14°	14.7	4.6	2.4	0.6

**Table 2:** NH_3_-sensing properties of the present work and reported in the literature.

Material	Method of Deposition	Limit of Detection	Operating Temperature (°C)	Reference
Pd–WO_3_ films	Spray pyrolysis	10 ppm	225	[9]
Ag–WO_3_ nanorods	Hydrothermal	50 ppb	450	[12]
WO_3_ nanoflakes	Spray Pyrolysis	120 ppm	150	[36]
V–WO_3_ films	Soft-chemical route	100 ppm	300	[37]
Cu–WO_3_ films	Soft-chemical route	100 ppm	200	[37]
Cr–WO_3_ nanosheets	Acidification with impregnation	2 ppm	400	[38]
WO_3_–Fe_2_O_3_ nanocomposites	Hydrothermal	25 ppm	300	[39]
Ag–WO_3_ films	Spray pyrolysis	500 ppb	200	This work

## Data Availability

The data presented in this study are available on request from the corresponding author.
